# Supplementary material for: iPS cell generation-associated point mutations include many C > T substitutions via different cytosine modification mechanisms
Source: Nat Commun. 2024 Jun 11;15:4946. doi: 10.1038/s41467-024-49335-5 (PMC11166658; doi:10.1038/s41467-024-49335-5)
Supplement: Supplementary file 1 — Supplementary Information [file 41467_2024_49335_MOESM1_ESM.pdf]

iPS cell generation-associated point mutations include many C>T substitutions via different cytosine modification mechanisms

Ryoko Araki, Tomo Suga, Yuko Hoki, Kaori Imadome, Misato Sunayama, Satoshi Kamimura, Mayumi Fujita and Masumi Abe

1. Supplementary Tables pg. 2
2. Supplementary Figures pg. 7
3. Supplementary References pg. 19

Supplementary Table 1

	**HipSci iPSCs** n=53	**E-HDFa iPSCs** n=5	**R-HDFa iPSCs** n=3	**CB-epi iPSCs** n=14	**germline mutations** n=816
total SNVs	2383.8 ± 2432.2 [128,727]	710.0 ± 135.6 [3,550]	461.3 ± 23.1 [1,384]	102.1 ± 21.2 [1,430]	- -
SNVs	2283.5 ± 2301.9 [121,024]	655.2 ± 135.6 [3,276]	434.3 ± 30.9 [1,303]	97.1 ± 23 [1,359]	44 [35,793]
CpG C>T	109.3 ± 115.8 [5,793]	40.6 ± 9.5 [203]	34.7 ± 4.6 [104]	15.4 ± 3.5 [215]	8 [6,390]
CpG C>T in CGI shore (<5Kb)	9.1 ± 8.8 [483]	3.2 ± 1.8 [16]	2.3 ± 0.6 [7]	1.5 ± 1.2 [21]	0.9 [728]
CpG C>T in CGI	1.9 ± 2.4 [102]	0.6 ± 1.3 [3]	0.3 ± 0.6 [1]	0.1 ± 0.3 [1]	0.1 [91]

Supplementary Table 1. Summary of iPSCs and germline mutations
Numbers of SNVs per cell line or individual are shown (mean ± SD).
The total number of mutations in each category detected in each type of iPSCs or germline is shown in parentheses.
Note that sex chromosomes were excluded from this comparative analysis because information on sex chromosome germline mutations is not available.

Supplementary Table 2

CpG C>T

	iPSCs	germline
LINE	1045	856
SINE	1614	1172
LTR	626	672
others	3030	3690
total	6315	6390

non-CpG C>T

	iPSCs	germline
LINE	12494	1532
SINE	6313	968
LTR	6658	956
others	29401	5433
total	54866	8889

other SNVs

	iPSCs	germline
LINE	15657	4030
SINE	7187	1969
LTR	7128	2238
others	35808	12277
total	65780	20514

Supplementary Table 2. **The numbers of mutations on LINE, SINE and LTR-retrotransposons**

Supplementary Table 3

Validated Variant rsID	Alleles	Variant Class	retrotransposon	Target Gene	Associated Disease
rs17133807	G/A	Cis Regulatory Element	-	IKZF1	Down +U2:U43Syndrome|Precursor Cell Lym
rs7430477	C/A/T	Cis Regulatory Element	-	Unclear	Atrial Fibrillation
rs849135	G/A	Cis Regulatory Element	-	Unclear	Diabetes Mellitus, Type 2
rs953413	G/A/C	Cis Regulatory Element	-	ELOVL2	Polyunsaturated Fatty Acid Metabolism
rs1026364	G/A/T	non-coding RNA	-	USF3	Osteoporosis
rs10036748	C/A/T	Cis Regulatory Element	LINE	TNIP1	Lupus Erythematosus, Systemic
rs9888739	C/T	Cis Regulatory Element	-	ITGAM	Lupus Erythematosus, Systemic
rs2107595	G/A	Cis Regulatory Element	-	HDAC9	Stroke
rs1690789	C/A/T	Cis Regulatory Element	-	TGFB2	Emphysema
rs35705950	C/A/T	Promoter	-	MUC5B	Idiopathic Pulmonary Fibrosis
rs2513789	G/A	Cis Regulatory Element	-	CDH17	Asthma
rs2446824	C/T	Cis Regulatory Element	-	CDH17	Asthma
rs402710	C/T	Cis Regulatory Element	-	TERT	Carcinoma
rs356165	G/A	Cis Regulatory Element	LINE	SNCA	Parkinson Disease
rs4265380	C/T	Cis Regulatory Element	-	RUNX3	Spondylitis, Ankylosing
rs4321755	C/T	Cis Regulatory Element	-	MRPS30	Carcinoma
rs4562997	G/A/T	Cis Regulatory Element	-	SMAD3	Thyroid Cancer, Papillary
rs36115365	G/A/C/T	Cis Regulatory Element	-	TERT	Carcinoma
rs7647481	G/A	Cis Regulatory Element	-	PPARG	Diabetes Mellitus, Type 2
rs35705950	G/A/T	Promoter	-	MUC5B	Idiopathic Pulmonary Fibrosis
rs3760511	G/A/T	Promoter	-	HNF1B	Ovarian Neoplasms
rs11655237	C/T	non-coding RNA	-	LINC00673	Pancreatic Neoplasms
rs2281721	C/T	Cis Regulatory Element	LTR	GALNT2	Cholesterol, HDL
rs73001406	G/A	Cis Regulatory Element	-	PHLDB1	Carcinoma|Glioma
rs73001406	G/A	Cis Regulatory Element	-	DDX6	Carcinoma|Glioma
rs10811656	C/T	Cis Regulatory Element	-	CDKN2A	Coronary Artery Disease
rs17716310	C/A/T	Cis Regulatory Element	-	PITX1	Colorectal Neoplasms
rs2370615	C/T	Cis Regulatory Element	-	PAG1	Hypersensitivity
rs6590330	G/A/T	Cis Regulatory Element	LINE	ETS1	Lupus Erythematosus, Systemic
rs72664324	G/A	Cis Regulatory Element	-	PLPP3	Coronary Artery Disease
rs4245739	C/A/G	non-coding RNA	-	MDM4	Prostatic Neoplasms
rs140490	G/A/C/T	Promoter	SINE	UBE2L3	Autoimmune Diseases
rs8064454	C/A/G	Cis Regulatory Element	-	HNF1B	Endometrial Neoplasms
rs2168518	G/A	non-coding RNA	-	GOSR2	Coronary Artery Disease
rs143383	G/A	Promoter	-	GDF5	Osteoarthritis
rs17248720	C/T	Promoter	-	LDLR	Hyperlipoproteinemia Type Ii
rs11603334	G/A	Promoter	-	ARAP1	Diabetes Mellitus, Type 2
rs2981578	C/A/T	Cis Regulatory Element	-	FGFR2	Breast Neoplasms
rs356219	G/A	Cis Regulatory Element	-	RP11-115D19.1	Dementia|Parkinson Disease
rs6495309	C/A/T	Cis Regulatory Element	LINE	CHRNA3	Lung Neoplasms|Tobacco Use|Tobacco Use
rs6776158	G/A	Promoter	SINE	CASR	Nephrolithiasis
rs143383	G/A	Cis Regulatory Element	-	GDF5	Osteoarthritis
rs554219	C/A/G/T	Cis Regulatory Element	LINE	CCND1	Breast Neoplasms
rs7705526	C/A/G/T	Cis Regulatory Element	SINE	TERT	Carcinoma|Hereditary Breast And Ovarian Ca
rs2736108	C/T	Promoter	-	TERT	Breast Neoplasms|Ovarian Neoplasms
rs4784227	C/A/T	Cis Regulatory Element	-	TOX3	Breast Neoplasms
rs2585175	G/A/C	Promoter	LTR	LY6K	Carcinoma
rs10750097	G/A/C	Promoter	-	APOA5	Cardiovascular Diseases
rs4132670	G/A	Cis Regulatory Element	-	TCF7L2	Diabetes Mellitus, Type 2
rs4072037	C/A/G/T	Promoter	-	MUC1	Stomach Neoplasms
rs143383	G/A	Promoter	-	GDF5	Osteoarthritis
rs6495309	C/A/T	Promoter	LINE	CHRNA3	Lung Neoplasms

Supplementary Table 3. Causal SNPs at CpG sites

We extracted 52 CpG C>T caused SNPs among 309 validated causal SNPs, which were reported previously[1] and showed the relationship with retrotransposons.

Supplementary Table 4

real-time PCR primers for expression analysis of human *TET1* gene

gene	forward primer (5' → 3')	reverse primer (5' → 3')
TET1	CAGGACCAAGTGTTGCTGCTGT	GACACCCATGAGAGCTTTTCCC
GAPDH	GTCTCCTCTGACTTCAACAGCG	ACCACCCTGTTGCTGTAGCCAA

PCR primers for confirmation of retroviral vector integration

constructs	forward primer (5' → 3')	reverse primer (5' → 3')
pMXs-Tet1	CCTTTTCGTGTGTCCCTTGT	TCTGTTCCTGACCTTGATCT
pMXs-c-Myc	GGA AACGACGAGAACAGTTG	TCTGTTCCTGACCTTGATCT
pMXs	GCATCGCAGCTTGGATACAC	TCTGTTCCTGACCTTGATCT
pMXs-Tet2	AACACAGGGTCTGTGACTAC	TCTGTTCCTGACCTTGATCT
pMXs-shRNA-Tet1	AAACCTAGTCTCCATGAGCC	TCTGTTCCTGACCTTGATCT
pMXs-shRNA-Tet2	CTTCTGGCAAACTTACATCC	TCTGTTCCTGACCTTGATCT

Target sequences of shRNA

target	Target Sequence(5'→3')	Reference
Tet1	GCTCATGGAGACTAGGTTTGG	Ito et al, 2010, Yu et al, 2015
Tet2	GGATGTAAGTTTGCCAGAAGC	Ito et al, 2010, Yu et al, 2015

Supplementary Table 4. shRNA sequences and primers used in this study

Supplementary Table 5

Human cells used for *TET1* gene expression analysis

somatic cells	source
adult human dermal fibroblasts (HDFa)	Lifeline Cell Technology, San Diego, CA USA, Lot#00967
adult human dermal fibroblasts	Lifeline Cell Technology, San Diego, CA USA, Lot#09212
adult human dermal fibroblasts	Thermo Fisher Scientific, Waltham, MA USA, Lot#1817891
adult human dermal fibroblasts	Thermo Fisher Scientific, Waltham, MA USA, Lot#2801264
adult human dermal fibroblasts	PromoCell, Heidelberg, Germany, Lot#495Z0274
CB-erythroblast-1	cord blood, single donor, ultra-pure, cryopreserved, PromoCell, Lot#406Z026
CB-erythroblast-2-1	cord blood, single donor, ultra-pure, cryopreserved, PromoCell, Lot#410Z009
CB-erythroblast-2-2	cord blood, single donor, ultra-pure, cryopreserved, PromoCell, Lot#409Z029
CB-erythroblast-2-3	cord blood, single donor, ultra-pure, cryopreserved, PromoCell, Lot#404Z0373

iPS cells
E-HDFa-1-3, establised from HDFa (Lot#00967)
E-HDFa-4-2, establised from HDFa (Lot#00967)
E-HDFa-7-3, establised from HDFa (Lot#00967)
E-HDFa-11-2, establised from HDFa (Lot#00967)
E-HDFa-12-3, establised from HDFa (Lot#00967)
R-HDFa-1-2, establised from HDFa (Lot#00967)
R-HDFa-1-12, establised from HDFa (Lot#00967)
R-HDFa-1-19, establised from HDFa (Lot#00967)
CB-epi-1-FF1, establised from CB-erythroblasts-1
CB-epi-1-1, establised from CB-erythroblasts-1
CB-epi-1-5, establised from CB-erythroblasts-1
CB-epi-1-8, establised from CB-erythroblasts-1
CB-epi-1-11, establised from CB-erythroblasts-1
CB-epi-2-1-3, establised from CB-erythroblasts-2-1
CB-epi-2-1-4, establised from CB-erythroblasts-2-1
CB-epi-2-1-5, establised from CB-erythroblasts-2-1
CB-epi-2-2-1, establised from CB-erythroblasts-2-2
CB-epi-2-2-3, establised from CB-erythroblasts-2-2
CB-epi-2-2-4, establised from CB-erythroblasts-2-2
CB-epi-2-3-1, establised from CB-erythroblasts-2-3
CB-epi-2-3-4, establised from CB-erythroblasts-2-3
CB-epi-2-3-7, establised from CB-erythroblasts-2-3

Supplementary Table 5. Human cells used for *TET1* gene expression analysis

Supplementary Fig. 1

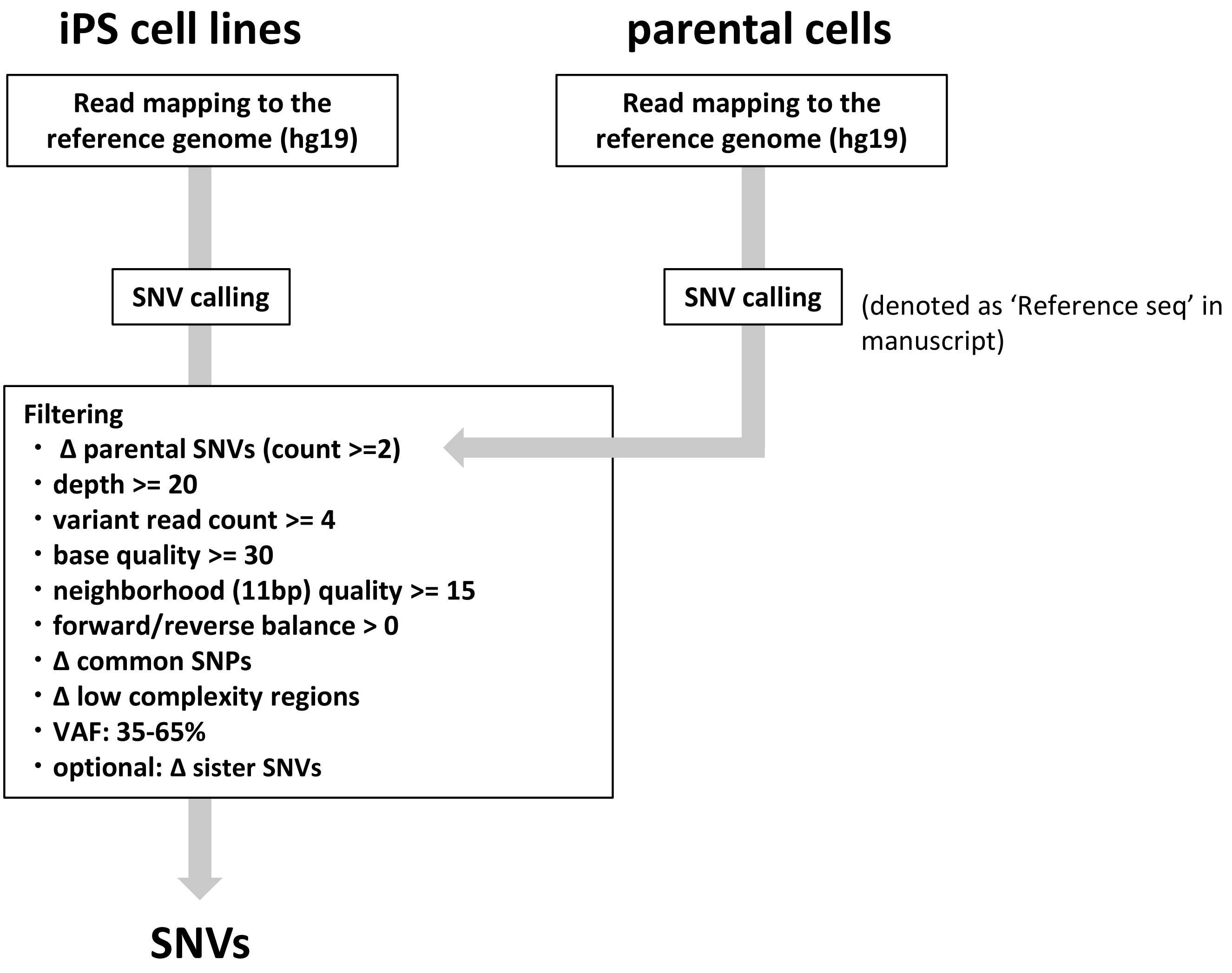

Supplementary Fig. 1 Informatics flow for identifying SNVs
The details of the mutation analysis of human iPSCs are shown. First, candidate SNVs of iPSCs and their parental cells are identified by mapping WGS data to the publicly available reference genome (hg19), and then candidate SNVs of iPSCs and their parental cells are compared using the filtering flow shown in this figure to identify iPSC-specific SNVs.

Supplementary Fig. 2

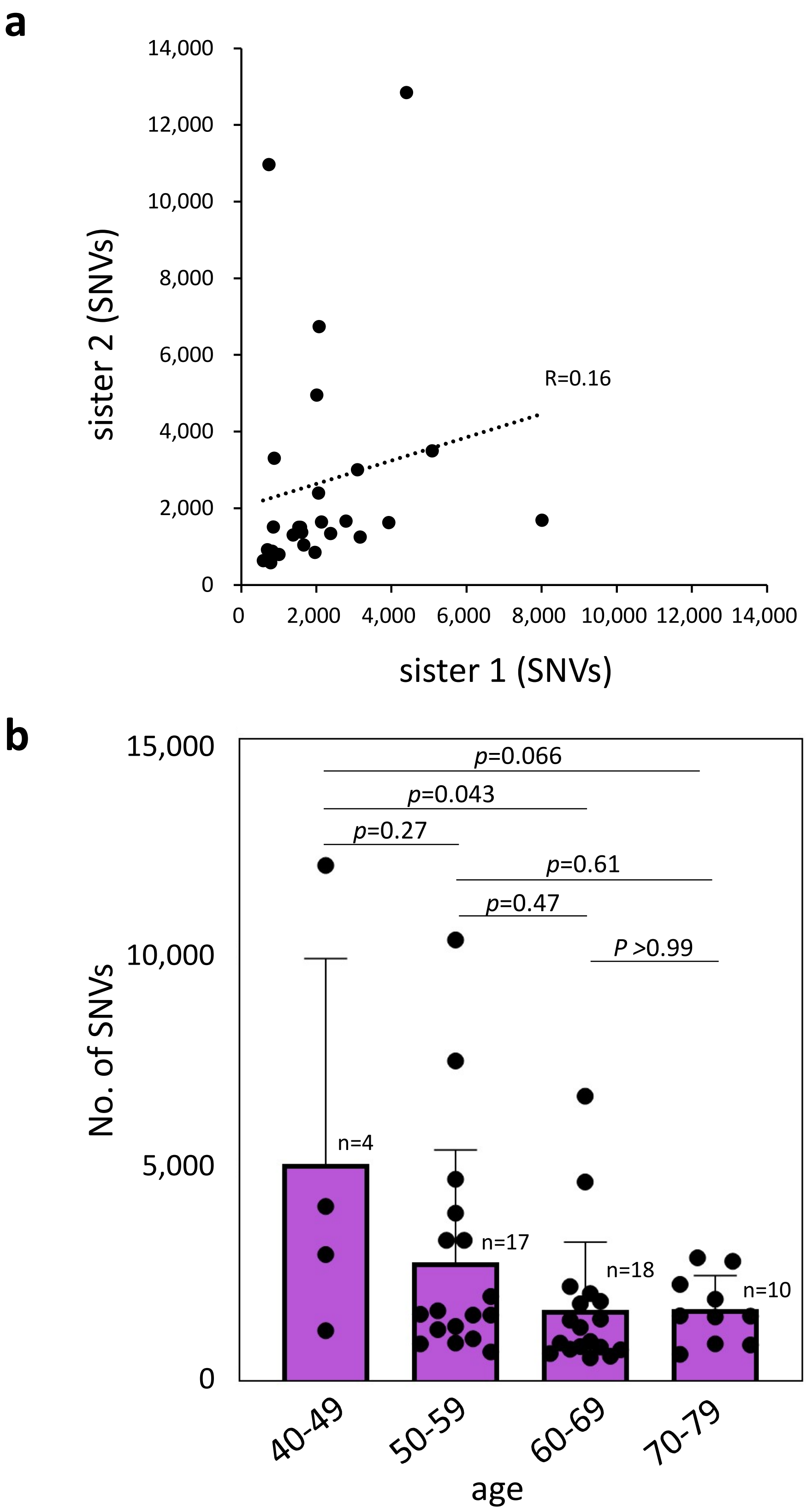

Supplementary Fig. 2 Mutation analysis of the iPSCs in the HipSci database

(a) Variation in the number of mutations between sister iPSCs

A low correlation (R=0.16) was revealed between the number of SNVs of two sister clones (sister 1 vs sister 2). We analysed 26 iPSC sister clones pairs (Fig. 2a).

(b) Relationship between donor age and the number of mutations identified in iPSC genomes

Error bars show the SD of the mean. The two-tailed one-way analysis of variance (ANOVA) was used for statistical analysis.

Source data are provided as a Source Data file.

Supplementary Fig. 3

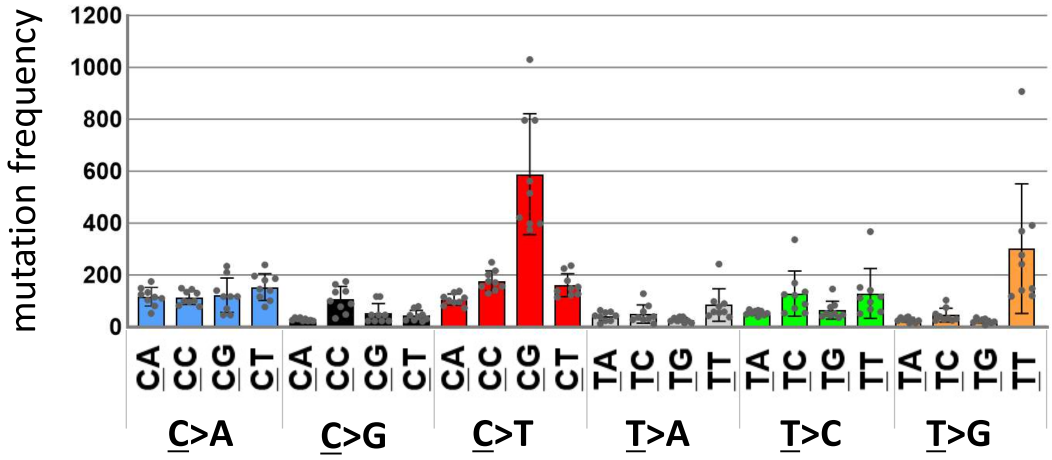

Supplementary Fig. 3 Mutational profiles of mouse iPSCs
Mutant bases were classified in 24 different ways in accordance with their 3'-neighbor bases. The Y-axis indicates the numbers of mutations normalized by the genomic abundance of their two contiguous bases, shown as number per 10^9 bases. Error bars show the SD of the mean (n=9 biological replicates).
Source data are provided as a Source Data file.

Supplementary Fig. 4

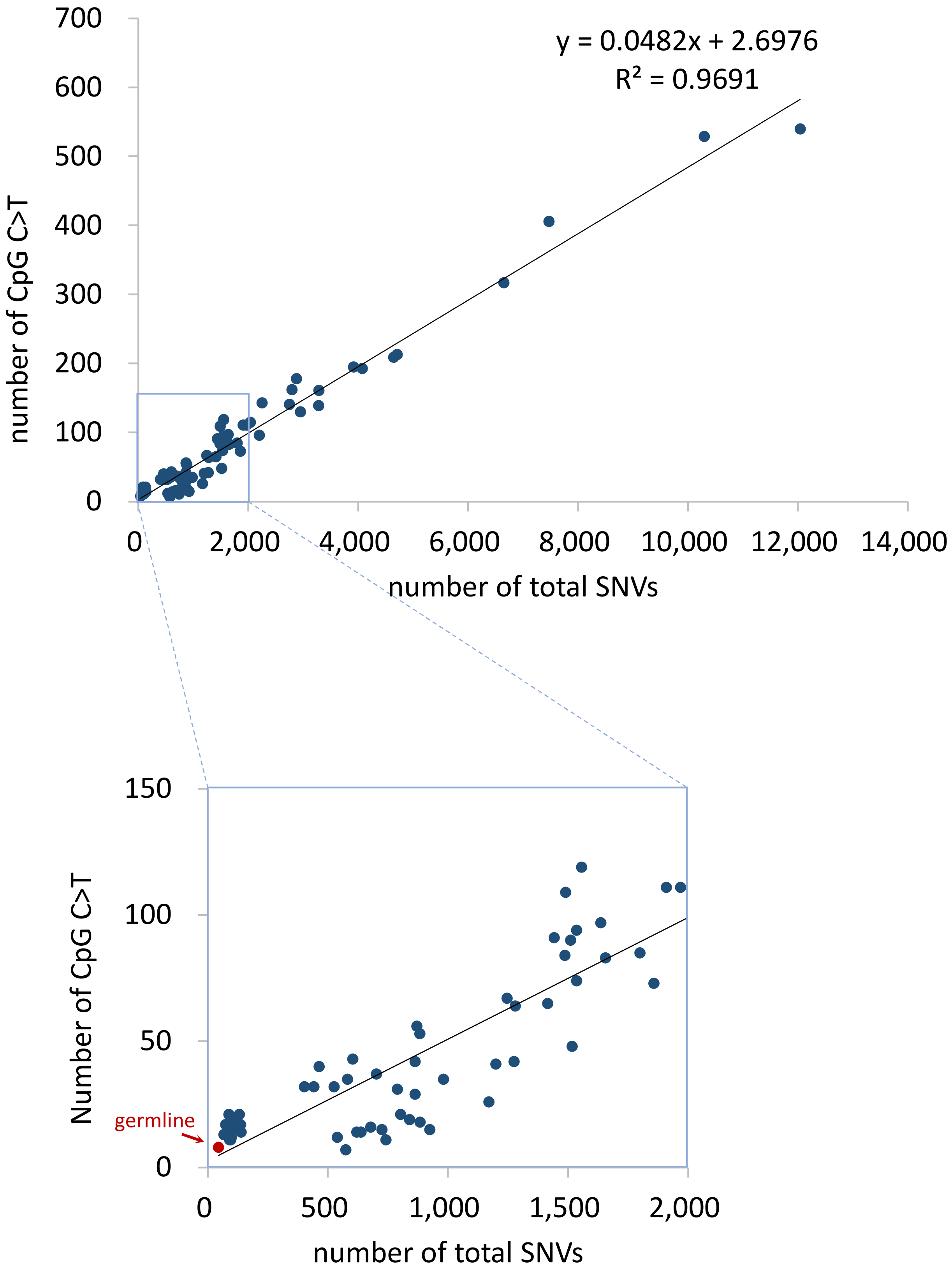

Supplementary Fig. 4 Correlation between total SNVs and CpG C>T mutations

Linear approximation was performed and the line is shown. A high correlation (indicated by the coefficient of determination R^2) was revealed between the number of SNVs and the CpG C>T transitions identified in iPSCs. Source data are provided as a Source Data file.

Supplementary Fig. 5

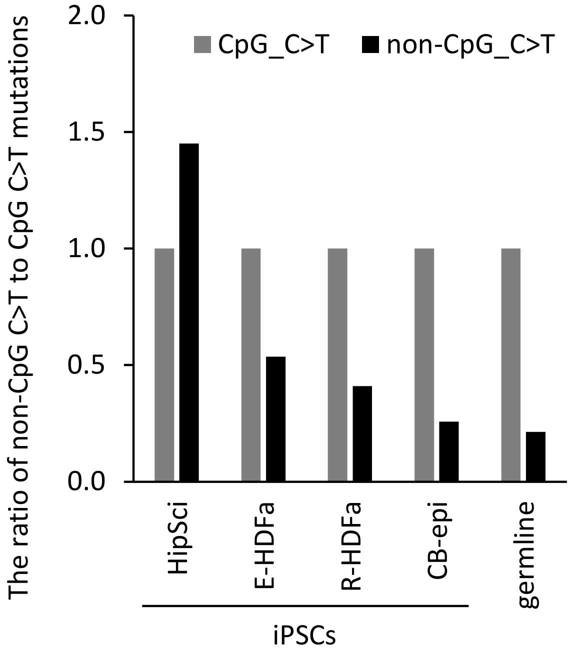

Supplementary Fig. 5 non-CpG C>T vs. CpG C>T
Source data are provided as a Source Data file.

Supplementary Fig. 6

a

	No. of colonies picked	No. of clones established*
shRNA-Tet1	8	3
shRNA-Tet2	8	1
shRNA-Tet1<br>shRNA-Tet2	8	0**

*iPSC Clones in which the shRNA of interest has been introduced.

**Two iPSC clones, in which only shRNA-Tet1 was integrated were established, but no clones integrated both.

b

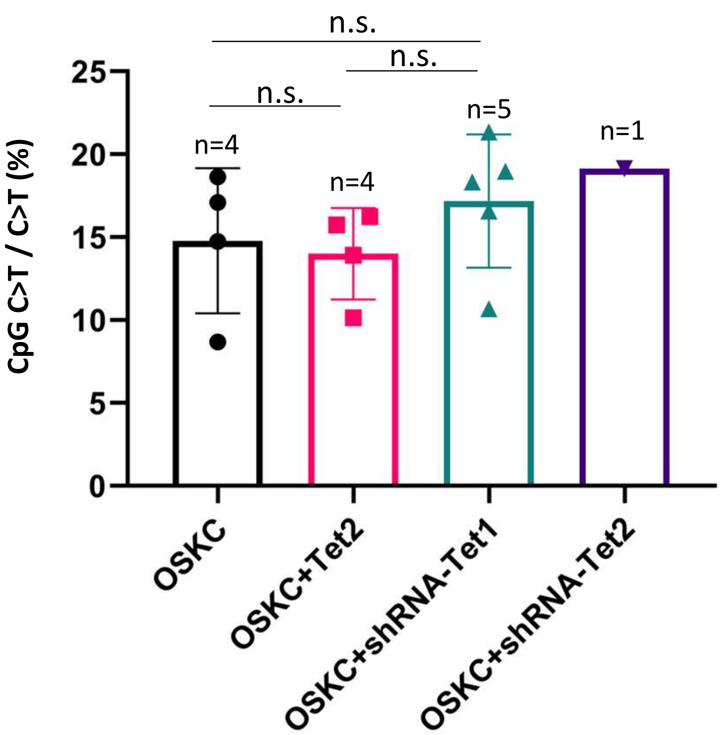

Supplementary Fig. 6 Forced expression of Tet2 and knockdown of Tet1 and Tet2 during iPSC generation
(a) Efficiency of shRNA-iPSC establishment
(b) Percentage of CpG C>T among C>T mutations
Error bars show the SD of the mean. For statistical analysis, the two-tailed Mann-Whitney-U test was employed. n.s.: not significant
Source data are provided as a Source Data file.

Supplementary Fig. 7

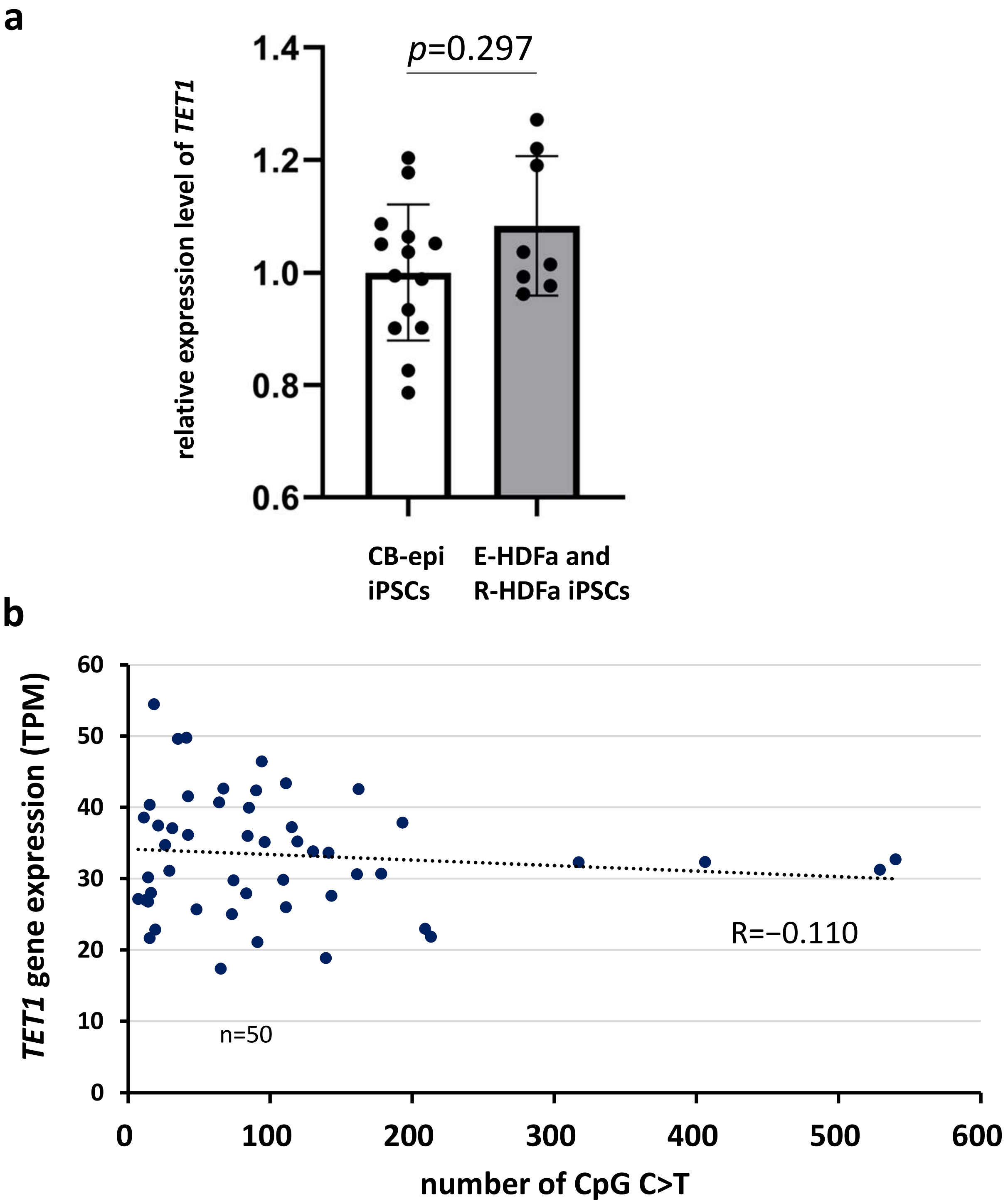

Supplementary Fig. 7 *TET1* gene expression in human iPSCs

(a) Relative expression level of the *TET1* gene in iPSCs (HDFa iPSCs vs. CB-epi iPSCs)

TET1 mRNA expression levels were assessed by RT-qPCR and data are normalized to *GAPDH* expression (CB-epi iPSCs, n=14 biological replicates; HDFa-iPSCs, n=8 biological replicates). Error bars show the SD of the mean. For statistical analysis, the Mann-Whitney-U test was employed. n.s., not significant.

(b) Number of CpG C>T mutations and *TET1* gene expression in HipSci iPSCs

The x-axis shows the number of CpG C>T mutations. The y-axis shows the expression level of *TET1* (European Nucleotide Archive, HipSci project). R indicates the correlation coefficient. Source data are provided as a Source Data file.

Supplementary Fig. 8

a

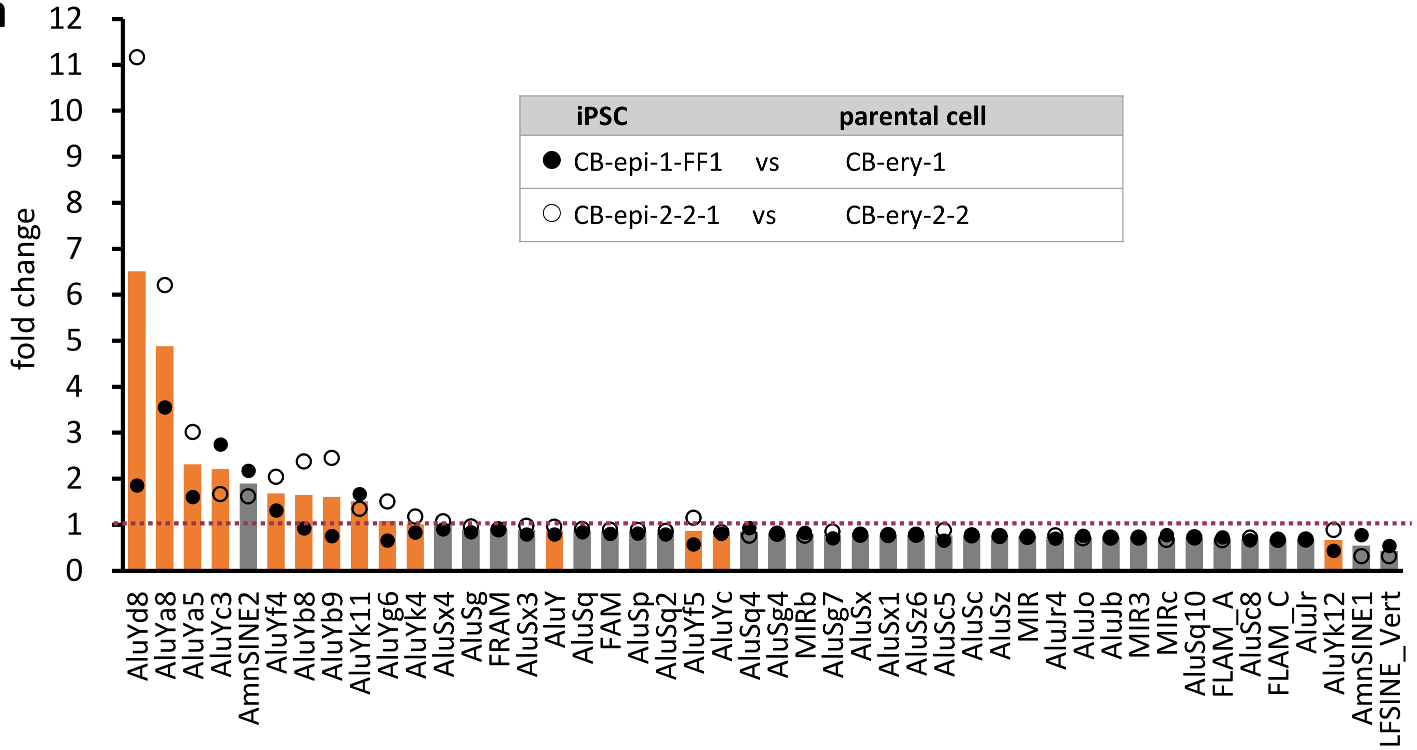

b

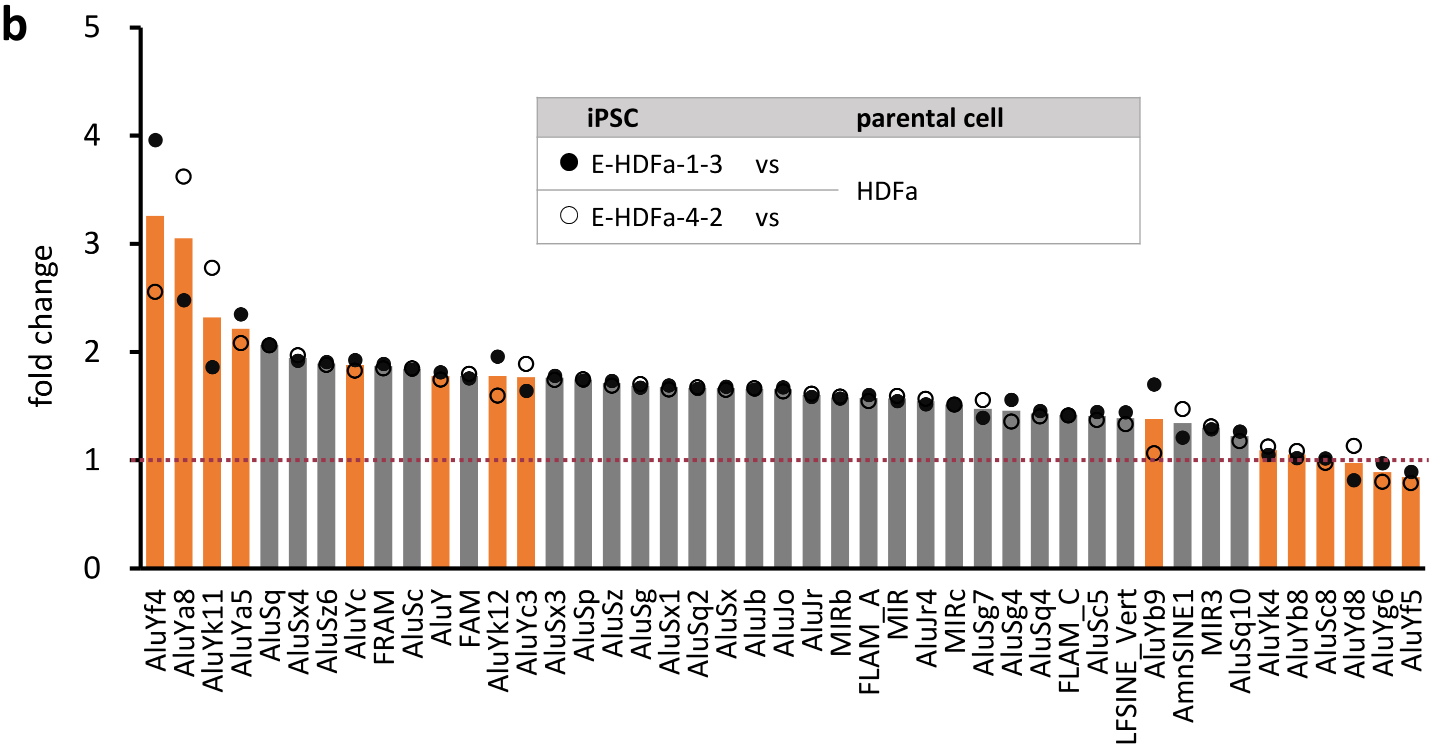

Supplementary Fig. 8 SINE expression in human iPSCs
(a) SINE expression in CB-epi-iPSCs compared with that in the parental cells
(b) SINE expression in HDFa-iPSCs (integration-free) compared with that in the parental cells
The x-axes show the subfamily name of the SINEs, and the y-axes indicate the fold change of expression in iPSCs relative to the parental cells. The bars show the average of the fold changes in the two iPSC lines examined. These values were calculated using TEtranscripts and DEseq2. Data from AluY and AluY subfamilies are shown in orange. SINE subfamilies with less than 5 reads in a sample were excluded from this graph.
Source data are provided as a Source Data file.

Supplementary Fig. 9

a

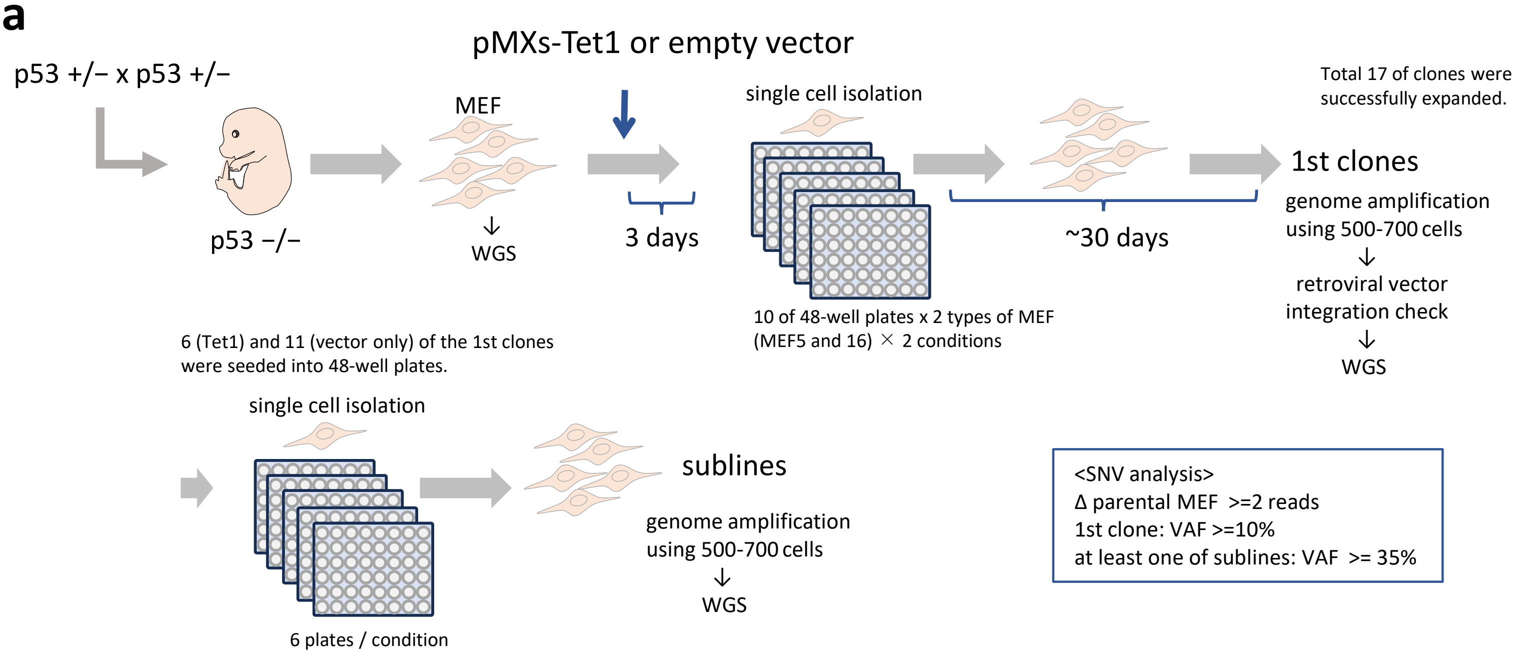

b

MEF	forced expression	1st clone	sublines
p53-/-MEF5	Tet1	p53M5-T1-3	p53M5-T1-3-1
			p53M5-T1-3-3
		p53M5-T1-5	p53M5-T1-5-1
			p53M5-T1-5-3
	vector	p53M5-V-25	p53M5-V-25-1
			p53M5-V-25-2
		p53M5-V-26	p53M5-V-26-3
			p53M5-V-26-4
p53-/-MEF16	Tet1	p53M16-T1-14	p53M16-T1-14-2
			p53M16-T1-14-3
		p53M16-T1-18	p53M16-T1-18-5
			p53M16-T1-18-6
	vector	p53M16-V-41	p53M16-V-41-2
			p53M16-V-41-3
		p53M16-V-42	p53M16-V-42-2
			p53M16-V-42-3

c

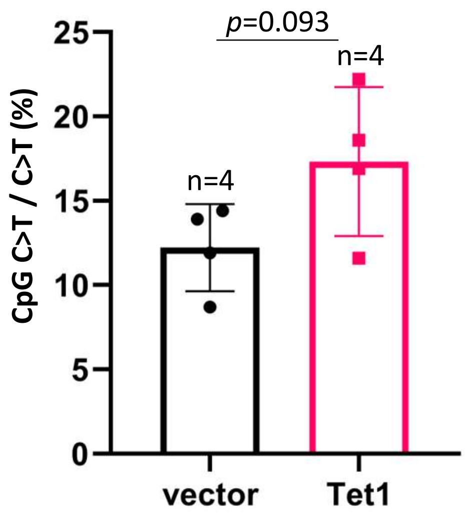

Supplementary Fig. 9 Effects of Tet1 on CpG C>T mutations in mouse p53−/− MEFs

(a) Experimental flow and SNV analysis

By applying a filter of at least 35% VAF in the subline, it is possible to reliably detect low frequency VAF mutations in the 1st clone.

(b) List of 1st clones and their sublines

Two independent male p53 −/− MEFs, MEF5 and MEF16, were used to establish these clones.

(c) Percentage of CpG C>T among C>T mutations

Error bars show the SD of the mean (n=4 biological replicates). The two-tailed *t*-test was used for statistical analysis.

Source data are provided as a Source Data file.

Supplementary Fig. 10

a

	HipSci iPSCs n=53	E-HDFa iPSCs n=5	R-HDFa iPSCs n=3	CB-epi iPSCs n=14	germline mutations n=816
SNVs in CDS	21.0±22.5 [1115]	7±1.2 [35]	3.7±2.5 [11]	0.7±0.6 [10]	0.8 [688]
CpG C>T in CDS	3.0±4.3 [158]	1.2±1.1 [6]	0.7±1.2 [2]	0.1±0.4 [2]	0.3 [254]

b

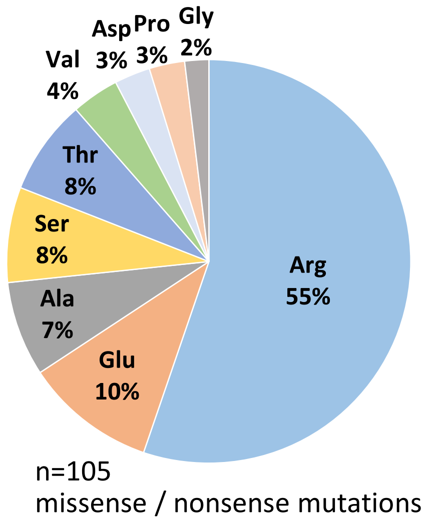

c

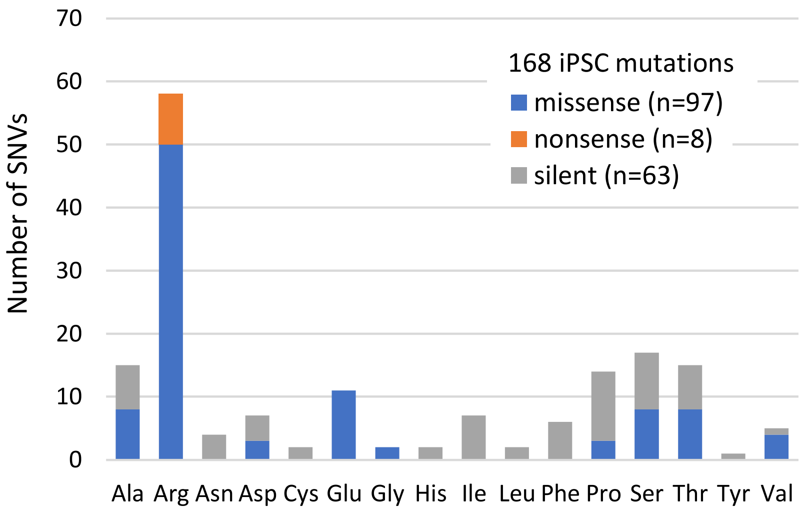

d

The Cancer Gene Census	Role in Cancer	a.a. substitution	iPSC line
BRIP1	tumor suppressor gene	p.Ala745Thr	tuju_4
SETD1B	tumor suppressor gene	p.Ser21Leu	zerv_8
TERT	oncogene, tumor suppressor gene	p.Thr839Met	deyz_3
ARID2	tumor suppressor gene	p.Pro10Leu	laey_4
ZFHX3	tumor suppressor gene	p.Ala779Val	oupf_6

Supplementary Fig. 10 Impact of CpG C>T mutations within CDS regions in iPSCs

(a) Number of total SNVs and CpG C>T mutations located in CDS regions in iPSCs

SNVs or CpG C>T transitions per cell line or individual is shown (mean ± SD). Total number of autosomal SNVs detected in each type of iPSCs or germline is indicated in parenthesis. Sex chromosomes were excluded from this comparative analysis because information on sex chromosome for germline mutations is not available.

(b, c) Amino acid substitutions resulting from CpG C>T mutations in iPSCs

(d) CpG C>T mutations identified in cancer-associated genes in iPSCs

The 736 genes listed in The Cancer Gene Census (CGC) were analyzed (https://cancer.sanger.ac.uk/census)[2].

Source data are provided as a Source Data file.

Supplementary Fig. 11

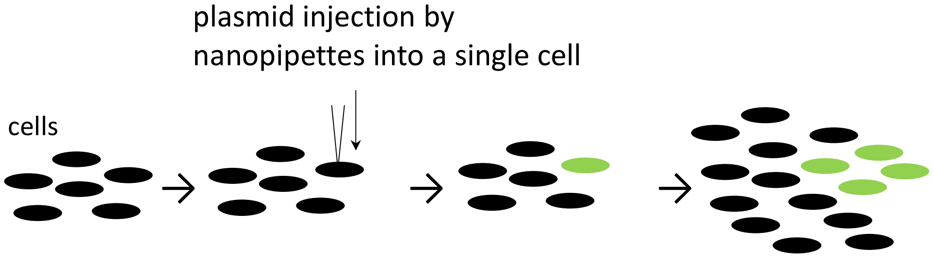

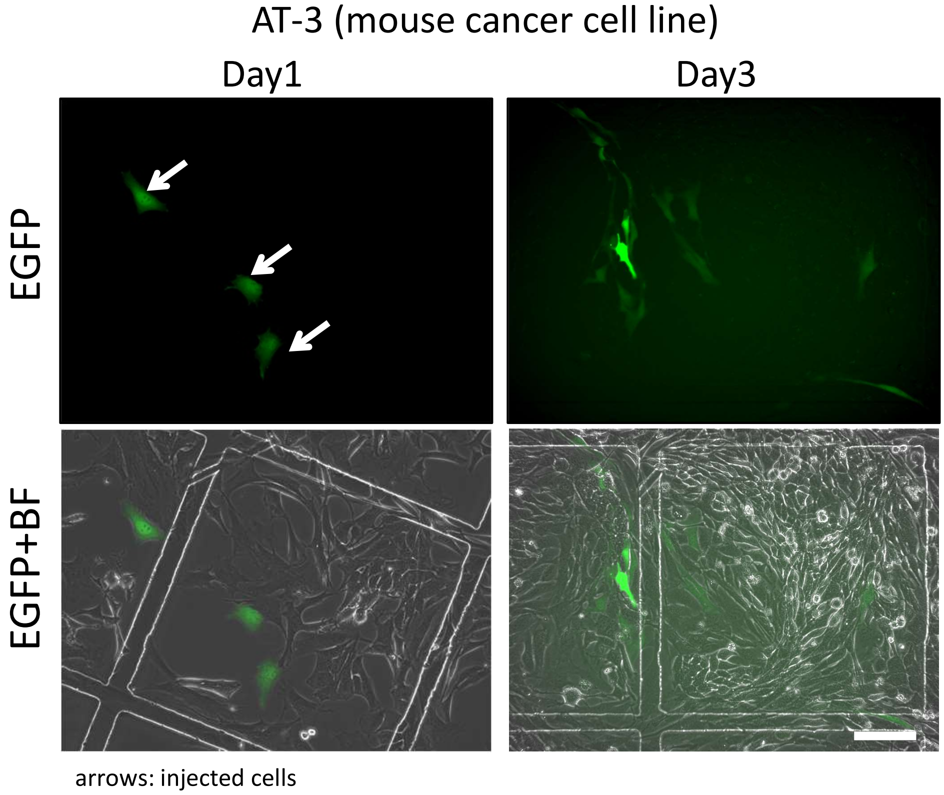

<Plasmid>
pEGFP-C1 (Clontech)
(40ng/ul)

<SU10 injection condition>
Approach Voltage: -0.5V
Current Drop Rate: 14%
Penetration Distance: 0.5 µm
Injection Voltage: -4 V
Injection Time: 0.2 s

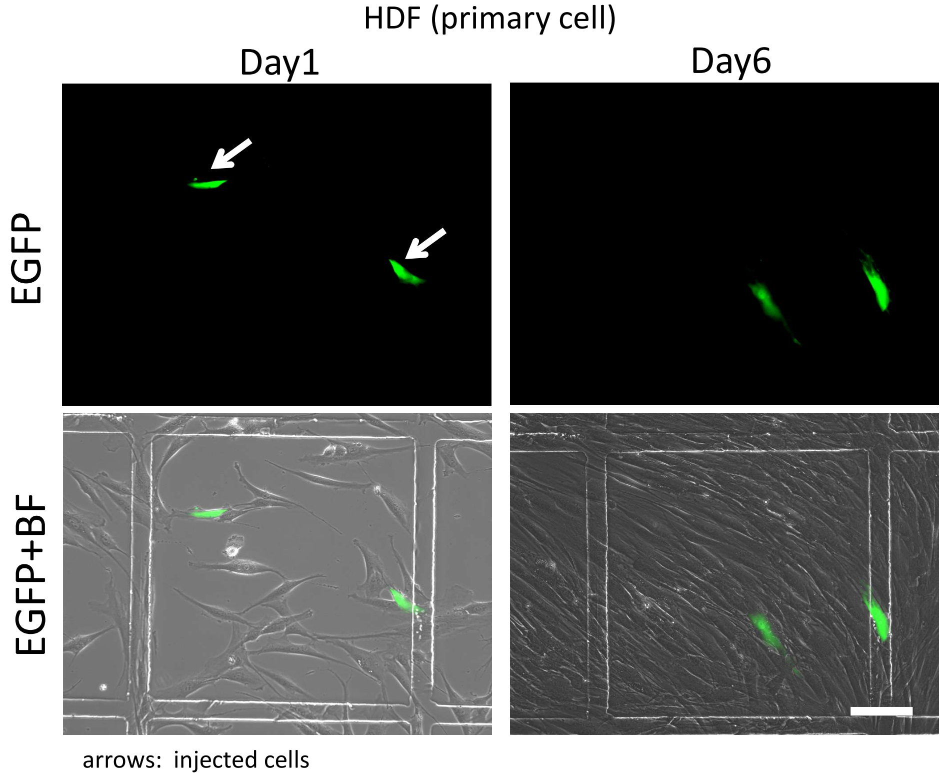

<Plasmid>
pEGFP-C1 (50ng/ul)

<SU10 injection condition>
Approach Voltage: -0.5 V
Current Drop Rate: 15%
Penetration Distance: 0.3 µm
Injection Voltage: -4 V
Injection Time: 0.5 s

Supplementary Fig. 11 Delivery of plasmid DNA into primary cells using nanopipettes
Single Cellome Unit SU10 (Yokogawa Electric Corporation, Tokyo, Japan) was used for this experiment [3].

Supplementary Fig. 12

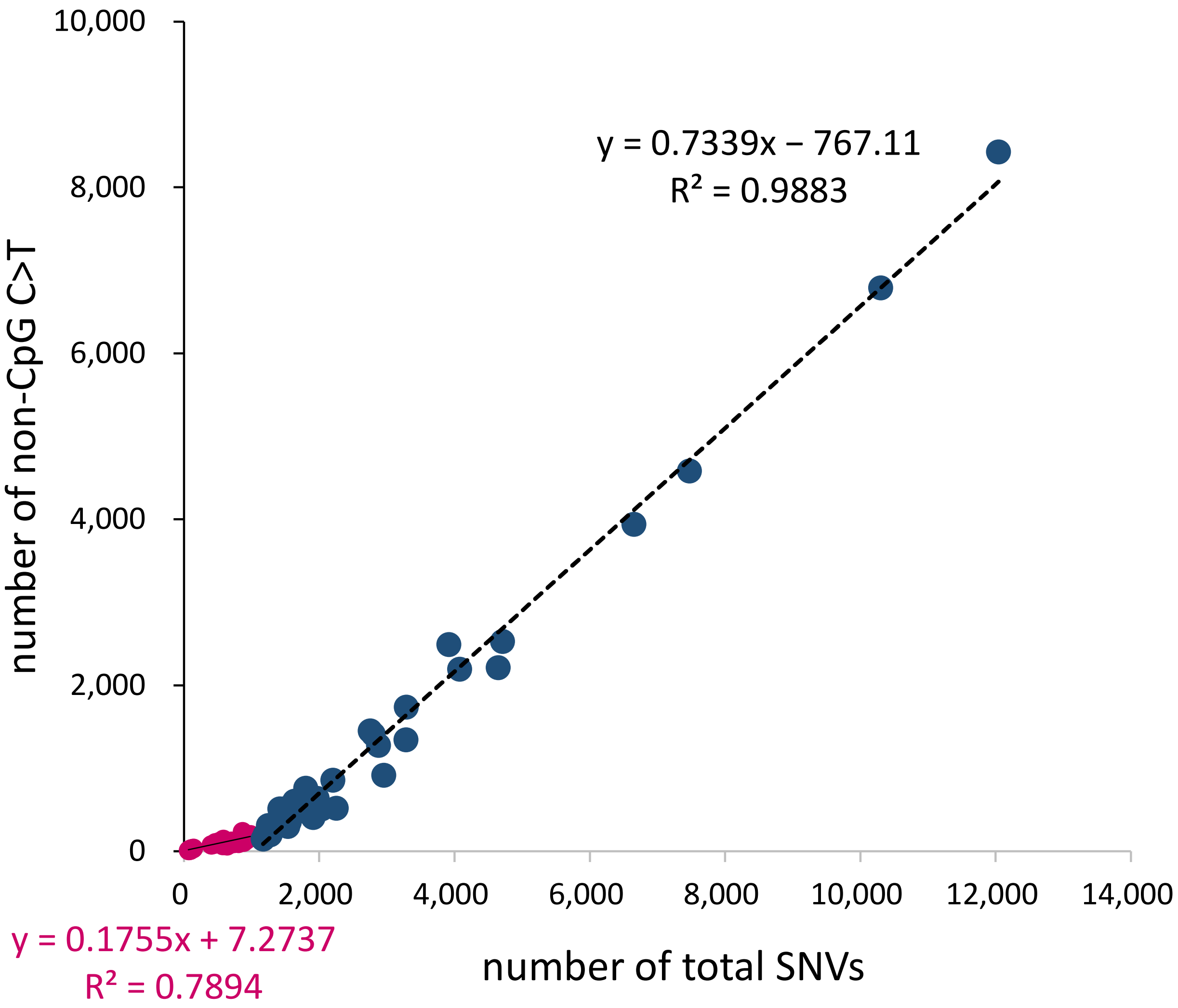

Supplementary Fig. 12 Relationship between total SNVs and non-CpG C>T mutations

High correlations (indicated by the coefficient of determination R^2) were revealed between the number of SNVs and the non-CpG C>T mutations. The mutation of C to T in sequences other than CpG showed biphasic. Source data are provided as a Source Data file.

Supplementary References

1. Alsheikh, A. J. *et al.* The landscape of GWAS validation; systematic review identifying 309 validated non-coding variants across 130 human diseases. *BMC Med Genomics* **15**, 74 (2022).
2. Sondka, Z. et al. The COSMIC Cancer Gene Census: describing genetic dysfunction across all human cancers. Nat Rev Cancer 18, 696-705 (2018).
3. Adam Seger, R. et al. Voltage controlled nano-injection system for single-cell surgery. Nanoscale 4, 5843-5846 (2012).
