# Supplementary material for: iPS cell generation-associated point mutations include many C > T substitutions via different cytosine modification mechanisms
Source: Nat Commun. 2024 Jun 11;15:4946. doi: 10.1038/s41467-024-49335-5 (PMC11166658; doi:10.1038/s41467-024-49335-5)
Supplement: Supplementary file 3 — Description of Additional Supplementary Information [file 41467_2024_49335_MOESM3_ESM.pdf]

Description of Additional Supplementary Information

File Name: Supplementary Data 1

Description: Quality information of WGS data and list of SNVs identified in iPSCs

File Name: Supplementary Data 2

Description: Amino acid substitutions caused by CpG C>T mutations in iPSCs
