# Supplementary material for: iPS cell generation-associated point mutations include many C > T substitutions via different cytosine modification mechanisms
Source: Nat Commun. 2024 Jun 11;15:4946. doi: 10.1038/s41467-024-49335-5 (PMC11166658; doi:10.1038/s41467-024-49335-5)
Supplement: Supplementary file 6 — Reporting Summary [file 41467_2024_49335_MOESM6_ESM.pdf]

nature portfolio

Corresponding author(s): Ryoko Araki, Masumi Abe

Last updated by author(s): Apr 24, 2024

Reporting Summary

Nature Portfolio wishes to improve the reproducibility of the work that we publish. This form provides structure for consistency and transparency in reporting. For further information on Nature Portfolio policies, see our Editorial Policies and the Editorial Policy Checklist.

Statistics

For all statistical analyses, confirm that the following items are present in the figure legend, table legend, main text, or Methods section.

n/a	Confirmed	
☐	☒	The exact sample size (*n*) for each experimental group/condition, given as a discrete number and unit of measurement
☐	☒	A statement on whether measurements were taken from distinct samples or whether the same sample was measured repeatedly
☐	☒	The statistical test(s) used AND whether they are one- or two-sided<br>*Only common tests should be described solely by name; describe more complex techniques in the Methods section.*
☒	☐	A description of all covariates tested
☒	☐	A description of any assumptions or corrections, such as tests of normality and adjustment for multiple comparisons
☐	☒	A full description of the statistical parameters including central tendency (e.g. means) or other basic estimates (e.g. regression coefficient) AND variation (e.g. standard deviation) or associated estimates of uncertainty (e.g. confidence intervals)
☐	☒	For null hypothesis testing, the test statistic (e.g. *F*, *t*, *r*) with confidence intervals, effect sizes, degrees of freedom and *P* value noted<br>*Give P values as exact values whenever suitable.*
☒	☐	For Bayesian analysis, information on the choice of priors and Markov chain Monte Carlo settings
☒	☐	For hierarchical and complex designs, identification of the appropriate level for tests and full reporting of outcomes
☒	☐	Estimates of effect sizes (e.g. Cohen's *d*, Pearson's *r*), indicating how they were calculated

Our web collection on statistics for biologists contains articles on many of the points above.

Software and code

Policy information about availability of computer code

Data collection	Illumina HiSeq X Ten, Novaseq6000 or MGI DNBseq-T7 sequencers<br>Keyence Biorevo BZ-X800
Data analysis	Basic alignment tool of CLC Genomics Workbench v12.0.3 (CLC Bio, Katrinebjerg, Denmark),<br>GraphPad Prism 8.4.3 (GraphPad Software)

For manuscripts utilizing custom algorithms or software that are central to the research but not yet described in published literature, software must be made available to editors and reviewers. We strongly encourage code deposition in a community repository (e.g. GitHub). See the Nature Portfolio guidelines for submitting code & software for further information.

Data

Policy information about availability of data

All manuscripts must include a data availability statement. This statement should provide the following information, where applicable:

- Accession codes, unique identifiers, or web links for publicly available datasets
- A description of any restrictions on data availability
- For clinical datasets or third party data, please ensure that the statement adheres to our policy

We used the whole genome sequencing data of human and mouse iPS cells which we established and analyzed.
Raw sequencing reads obtained in this study were registered in the DDBJ Sequence Read Archive (DRA) with the following accession codes: DRA015842,

DRA017394, DRA017395 and DRA017396.
The previously generated raw Illumina sequencing reads analyzed during the current study have also been deposited in the DRA under accession codes DRA002956, DRA006232, DRA006457, DRA006622, DRA007325, DRA007336, DRA008453, DRA008459 and DRA012278.
We also analyzed the whole genome sequencing data of human iPS cells generated by the HipSci Consortium, funded by The Wellcome Trust and the MRC (https://www.hipsci.org/).

Research involving human participants, their data, or biological material

Policy information about studies with human participants or human data. See also policy information about sex, gender (identity/presentation), and sexual orientation and race, ethnicity and racism.

Reporting on sex and gender	NA
Reporting on race, ethnicity, or other socially relevant groupings	NA
Population characteristics	NA
Recruitment	NA
Ethics oversight	NA

Note that full information on the approval of the study protocol must also be provided in the manuscript.

Field-specific reporting

Please select the one below that is the best fit for your research. If you are not sure, read the appropriate sections before making your selection.

☒ Life sciences ☐ Behavioural & social sciences ☐ Ecological, evolutionary & environmental sciences

For a reference copy of the document with all sections, see nature.com/documents/nr-reporting-summary-flat.pdf

Life sciences study design

All studies must disclose on these points even when the disclosure is negative.

Sample size	No specific methods were used for sample size estimation. Sample size was determined based on the literature.
Data exclusions	No data was excluded.
Replication	Mutation analysis<br>Human iPS cells: 75 biological replicates (14 Cord blood-episomal vector iPSCs, 5 dermal fibroblast-epismal vector iPSCs, 3 dermal fibroblast-retrovirus vector iPSCs, 53 dermal fibroblast-Sendai virus vector iPSCs)<br>Mouse iPS cells:<br>1) 3 (control iPSCs) or 6 (Tet1-iPS cells) replicates<br>2) 4 (control iPSCs) or 4 (Tet2-iPS cells) replicates<br>3) 4 (control iPSCs) or 5 (shRNA-Tet1 iPS cells) replicates<br>p53-/-MEF:<br>4 (control) or 4 (Tet1-forced expression) replicates<br><br>Colony formation assay<br>MEF: one experiment with three technical replicates
Randomization	Not relevant to the study, as no animal or human subjects were involved in it.
Blinding	The data presented did not require the use of blinding. Data reported for these experiments were not subjective but rather based on quantitative analyses.

Reporting for specific materials, systems and methods

We require information from authors about some types of materials, experimental systems and methods used in many studies. Here, indicate whether each material, system or method listed is relevant to your study. If you are not sure if a list item applies to your research, read the appropriate section before selecting a response.

Materials & experimental systems

n/a	Involved in the study	
☒	☐	Antibodies
☐	☒	Eukaryotic cell lines
☒	☐	Palaeontology and archaeology
☐	☒	Animals and other organisms
☒	☐	Clinical data
☒	☐	Dual use research of concern
☒	☐	Plants

Methods

n/a	Involved in the study	
☒	☐	ChIP-seq
☒	☐	Flow cytometry
☒	☐	MRI-based neuroimaging

Eukaryotic cell lines

Policy information about cell lines and Sex and Gender in Research

Cell line source(s)	Human and mouse iPS cells and ntES cells were originally established in our labs.
Authentication	Cell lines were not externally authenticated.
Mycoplasma contamination	Sampling tests using PCR or whole genome sequencing data revealed that the cell lines used in this study are mycoplasma negative.
Commonly misidentified lines (See ICLAC register)	No commonly misidentified cell lines were used in this study.

Animals and other research organisms

Policy information about studies involving animals; ARRIVE guidelines recommended for reporting animal research, and Sex and Gender in Research

Laboratory animals	C57BL/6 (Japan SLC), Nanog-GFP tg [STOCK Tg (Nanog-GFP, puro) 1Yam] (RBRC#02290, RIKEN BRC) and B6.Cg-Trp53<tm1Sia>/Rbrc (RBRC01361, RIKEN BRC) were used for MEF preparation.
Wild animals	This study did not involve wild animals.
Reporting on sex	C57BL/6 (Japan SLC) and B6.Cg-Trp53 embryonic fibroblasts: male, Nanog-GFP tg embryonic fibroblasts: female
Field-collected samples	NA
Ethics oversight	All experiments adhered to institutional guidelines.

Note that full information on the approval of the study protocol must also be provided in the manuscript.
